# Supplementary material for: TNIK inhibition abrogates colorectal cancer stemness
Source: Nat Commun. 2016 Aug 26;7:12586. doi: 10.1038/ncomms12586 (PMC5007443; doi:10.1038/ncomms12586)
Supplement: Supplementary Information — Supplementary Figures 1-15 and Supplementary Tables 1-4, Supplementary Methods [file ncomms12586-s1.pdf]

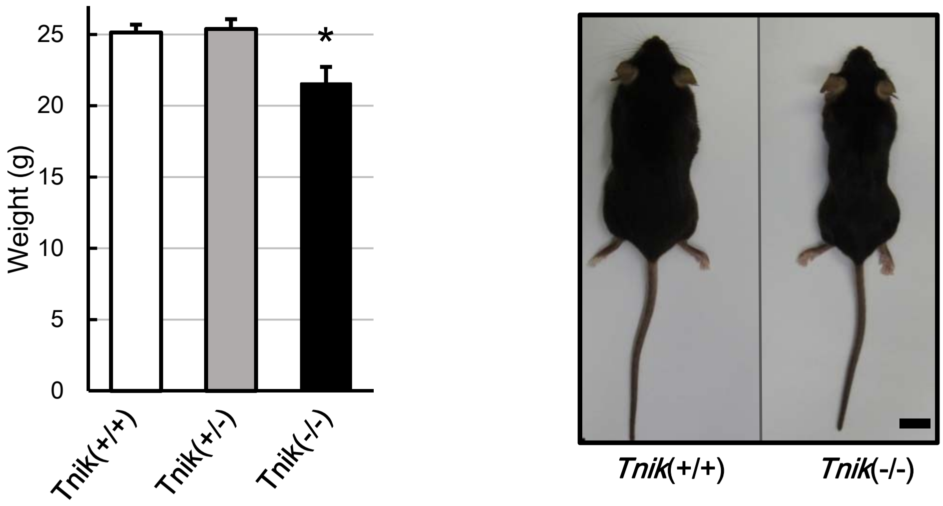

Supplementary Figure 1: Reduction of Body Weight in *Tnik*$^{-/-}$ Mice.
Body weight of 10-11-week-old male C57BL/6J-*Tnik*$^{+/+}$ (n = 5), *Tnik*$^{+/-}$ (n = 5), and *Tnik*$^{-/-}$ (n = 4) mice (left) and their representative appearance (right). Data are presented as mean ± s.e.m. *Significant difference with P-value of <0.05 relative to wild-type and heterozygous littermates. Scale bar, 1 cm.

a

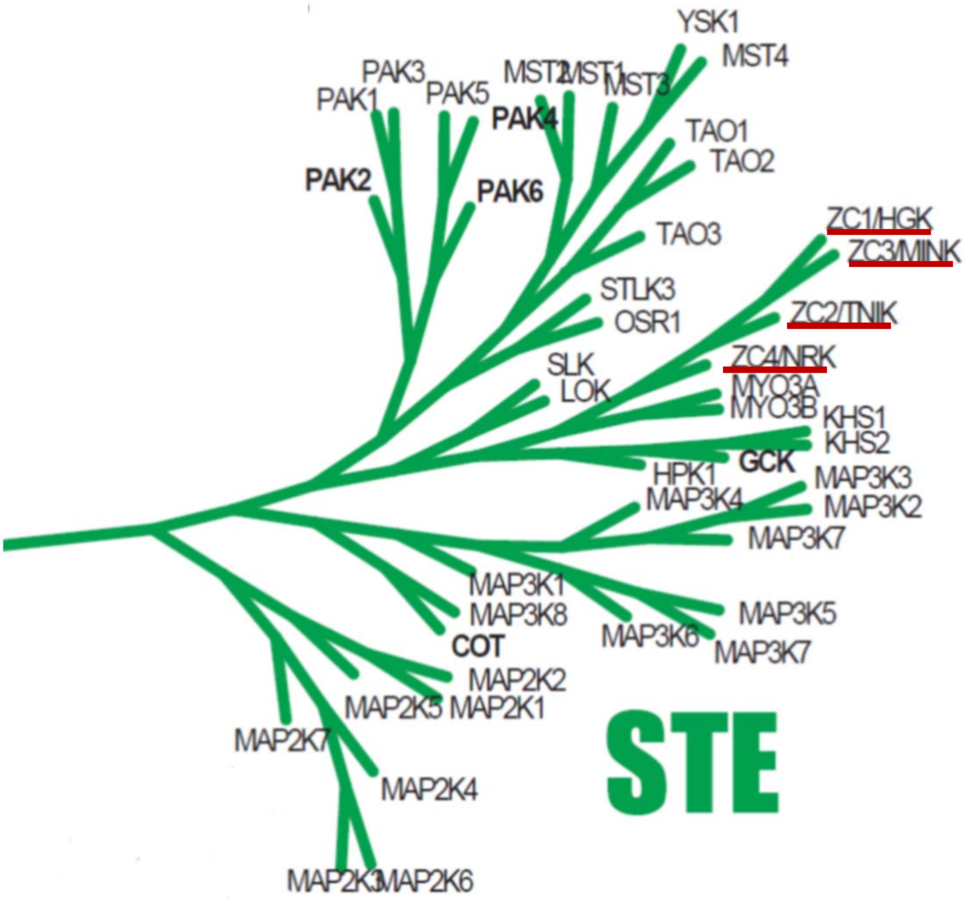

b

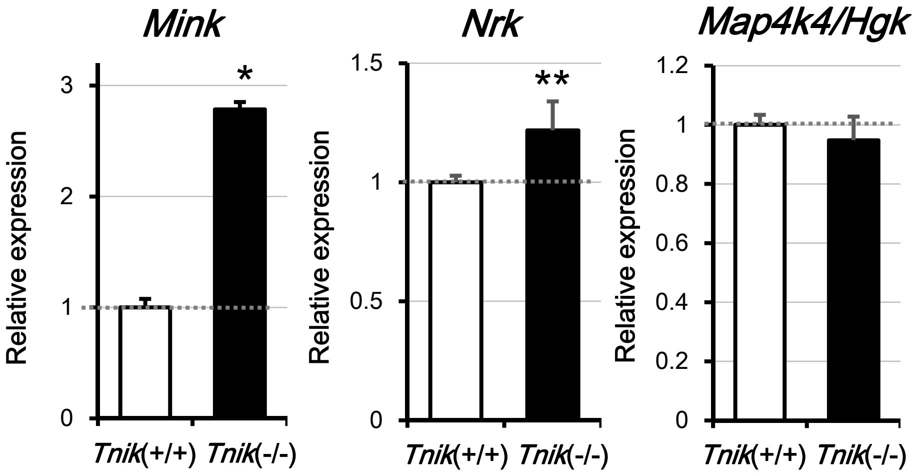

Supplementary Figure 2: Compensatory Up-regulation of *Mink* and *Nrk*.
(**a**) Phylogenetic tree of STE20 family kinases. Kinases closely related to TNIK are underlined.
(**b**) Relative expression of the *Mink* (ZC3), *Nrk* (ZC4), and *Map4k4* (*Hgk*) (ZC1) genes in MEFs derived from $Tnik^{+/+}$ (set to 1) and $Tnik^{-/-}$ mice. Data are presented as mean ± s.e.m. $^{**}P < 0.01$; $^{*}P < 0.05$.

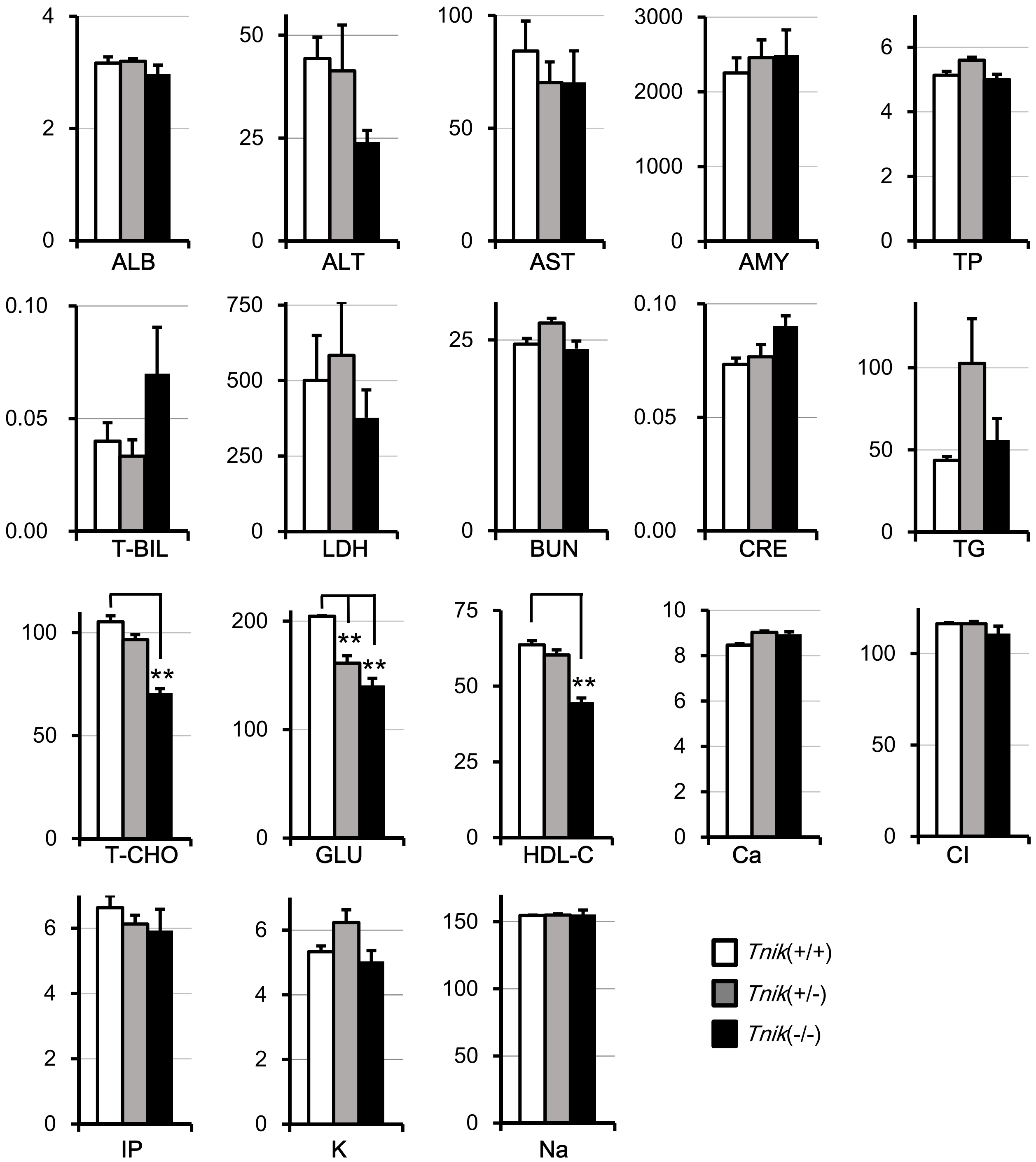

Supplementary Figure 3: Serum Biochemical Analysis of *Tnik*$^{-/-}$ Mice.
Abbreviations: ALB, albumin (g/dL); ALT, alanine aminotransferase (IU/L); AST, aspartate aminotransferase (IU/L); AMY, amylase (IU/L); TP, total protein (g/dL); T-BIL, total bilirubin (mg/dL); LDH, lactate dehydrogenase (IU/L); BUN, blood urea nitrogen (mg/dL); CRE, creatinine (mg/dL); TG, triglyceride (mg/dL); T-CHO, total cholesterol (mg/dL); GLU, glucose (mg/dL); HDL-C, high-density lipoprotein cholesterol (mg/dL); Ca, calcium (mg/dL); Cl, chloride (mEq/L); IP, inorganic phosphorus (mg/dL); K, potassium (mEq/L); Na, sodium (mEq/L).

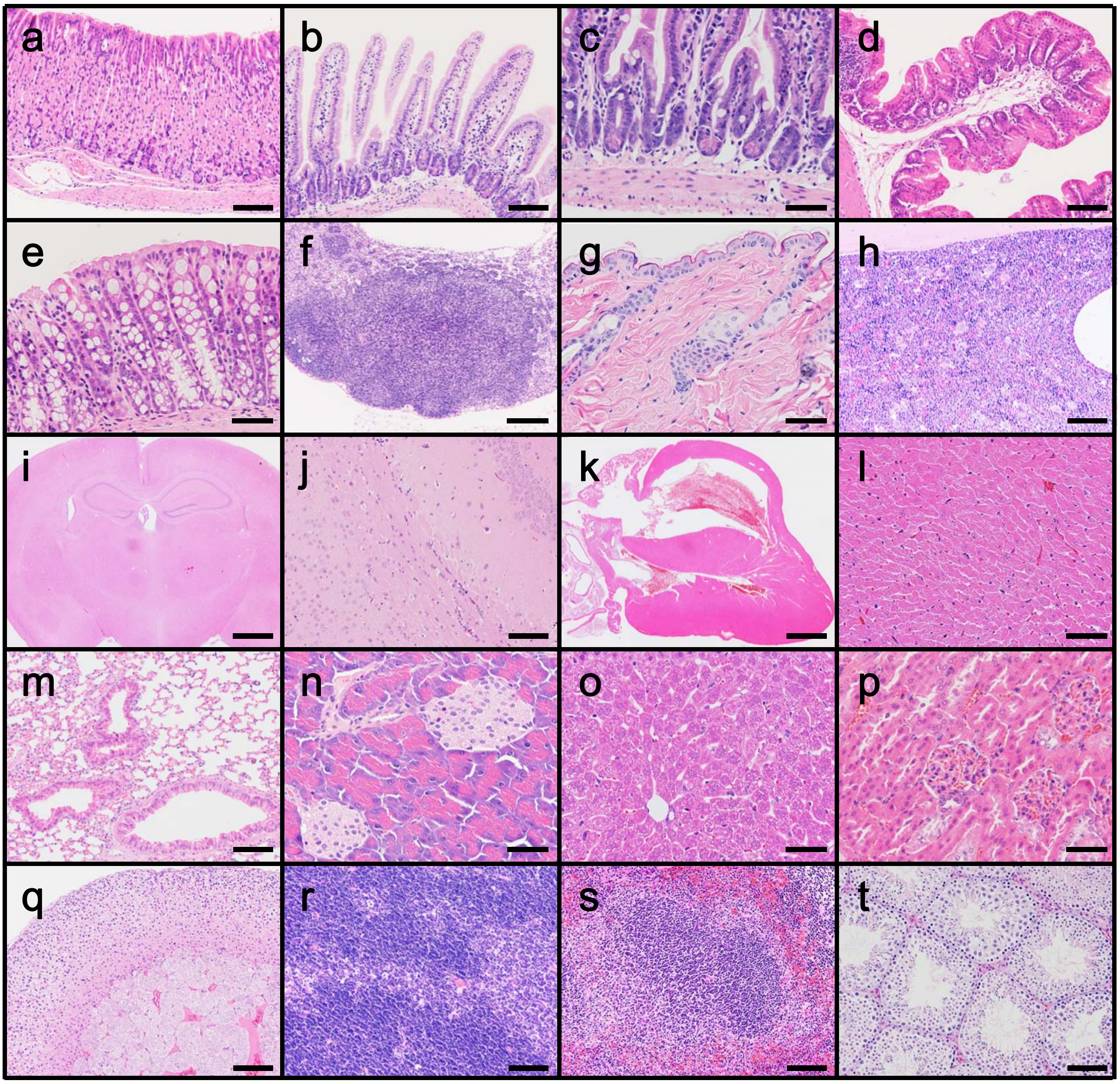

Supplementary Figure 4: Representative Organ Histology of *Tnik*$^{-/-}$ Mice. Histology (hematoxylin and eosin staining) of the stomach (**a**), small intestine (**b** and **c**), colon (**d**), rectum (**e**), mesenteric lymph node (**f**), skin (**g**), bone marrow (**h**), brain (**i** and **j**), heart (**k** and **l**), lung (**m**), pancreas (**n**), liver (**o**), kidney (**p**), adrenal gland (**q**), thymus (**r**), spleen (**s**), and testis (**t**) of a 10-week old male *Tnik*$^{-/-}$ mouse. Scale bars, 50 μm (**c**, **e**, **g**, **l**, and **n-p**); 100 μm (**a**, **b**, **d**, **h**, **j**, **m**, and **q-t**); 200 μm (**f**); 1 mm (**i** and **k**).

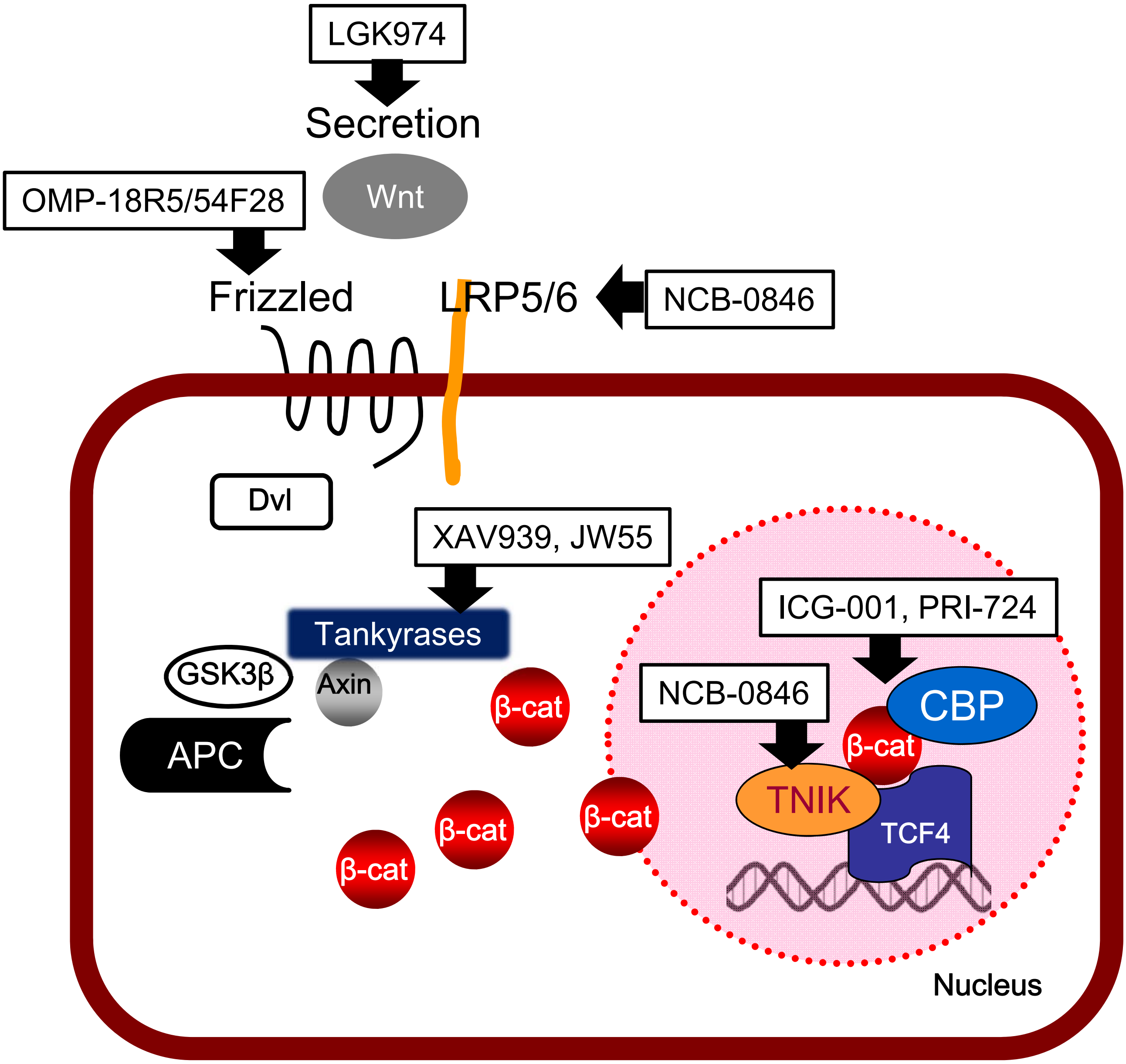

Supplementary Figure 5: Wnt Signaling Pathway in a Colorectal Cancer Cell with a Mutated *APC* Gene.

Therapeutics targeting various molecular components of the Wnt signaling pathway are illustrated: porcupine (LGK974), frizzled receptors (OMP-18R5 and OMP-54F28), tankyrases (XAV939 and JW55), CBP (ICG-001 and PRI-724), and TNIK (NCB-0846). The β-catenin (β-cat) destructive complex is not properly formed due to the truncational mutation of the *APC* gene.

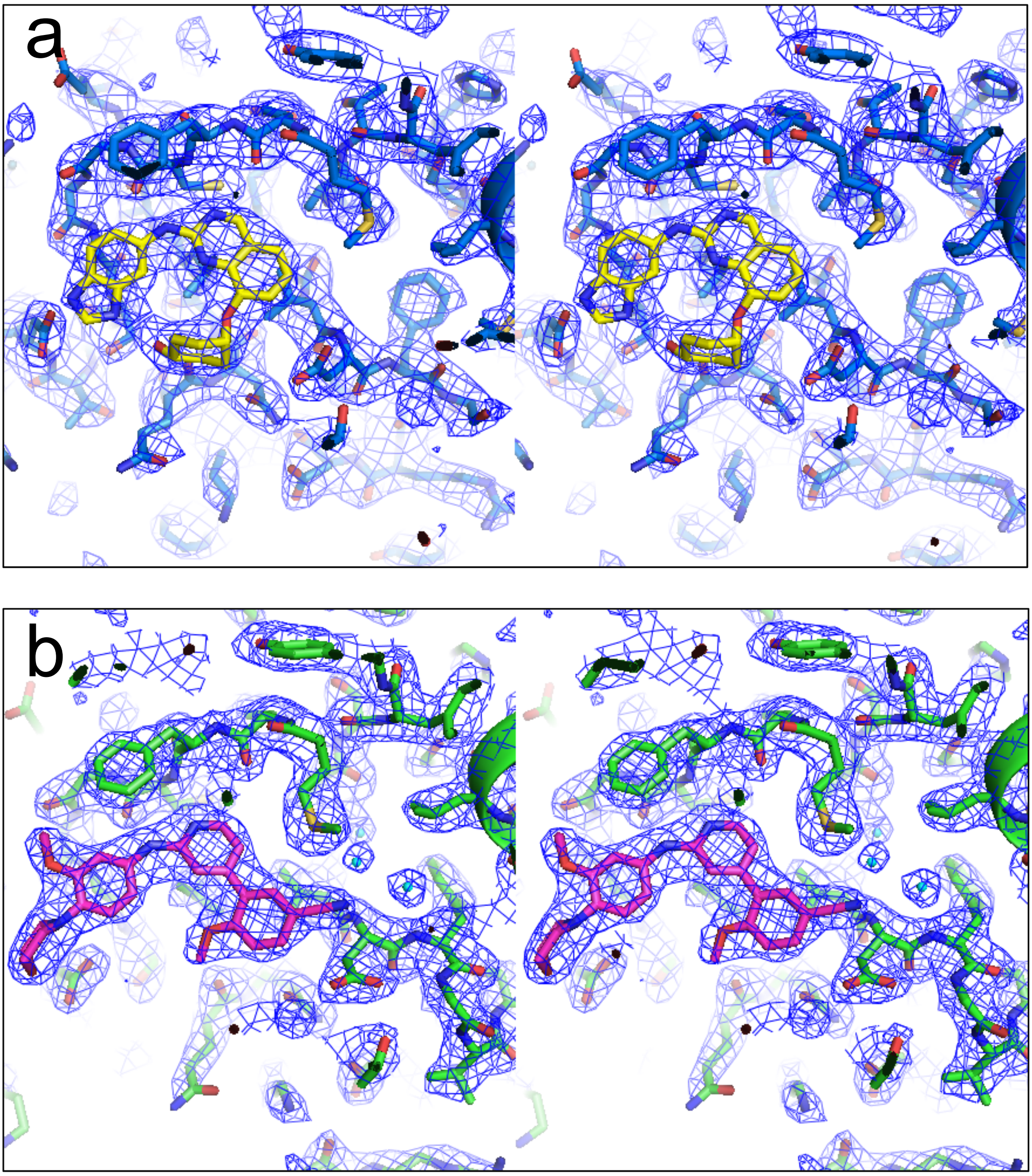

Supplementary Figure 6: Electron Density Map.
Stereo views of NCB-0846, Compound 9, and the DFG motif, shown together with the 2Fo-Fc electron density map (1σ). NCB-0846 (**a**), Compound 9 (**b**), and water molecules are shown as yellow and magenta sticks, and cyan balls, respectively.

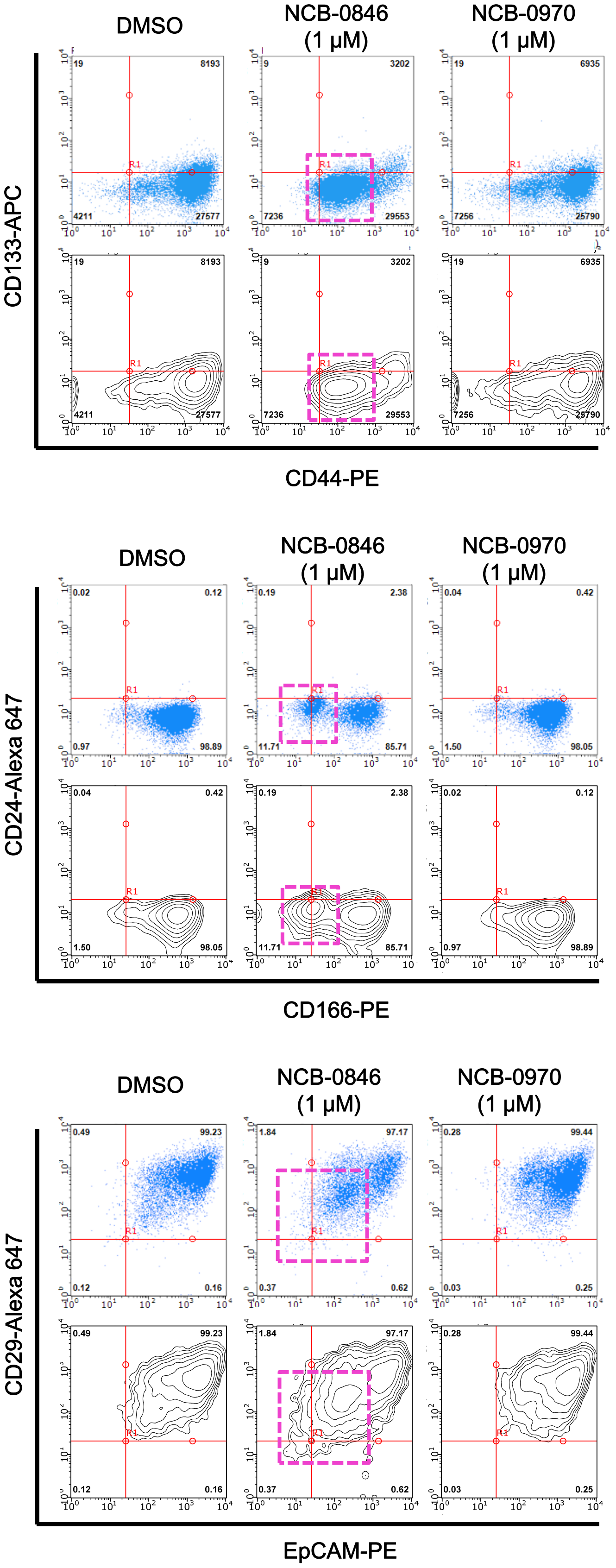

Supplementary Figure 7: Inhibition of CSC Marker Expression by NCB-0846. HCT116 cells were treated with DMSO (control), 1 µM NCB-0846, or 1 µM NCB-0970 for 96 hours. Cells expressing CSC markers (CD44/CD133, CD166/CD24, and CD29/EpCAM) were analyzed by flow cytometry. Cell populations with reduced expression of CSC markers are indicated by clear squares in magenta. Abbreviations: APC, allophycocyanin; PE, phycoerythrin.

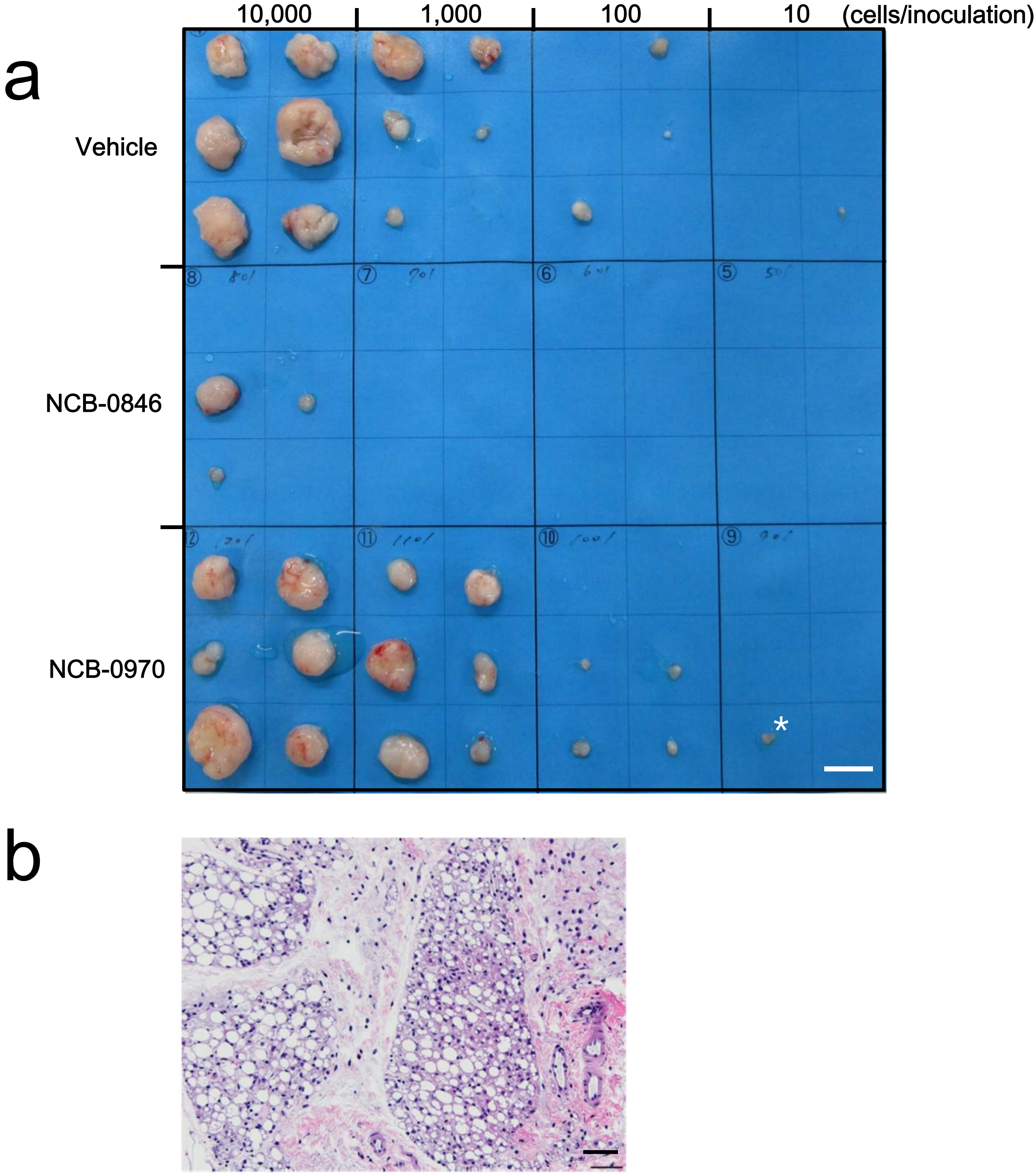

Supplementary Figure 8: Suppression of Tumor Formation by CSCs.
(**a**) DLD-1 cells were treated with DMSO, 1 µM NCB-0846, or 1 µM NCB-0970 for 96 hours. Ten thousand, 1000, 100, or 10 viable cells were injected into the flanks of 6-week-old male NOD/ShiJic-scid mice. Forty days later, the developed tumors were excised and photographed. Bar, 1.0 cm. Histology of the tumor marked with an asterisk is shown in (**b**).
(**b**) Histology of a subcutaneous tumor formed in a NOD/ShiJic-scid mouse inoculated with 10 DLD-1 cells pretreated with NCB-0970. Bar, 50 µm.

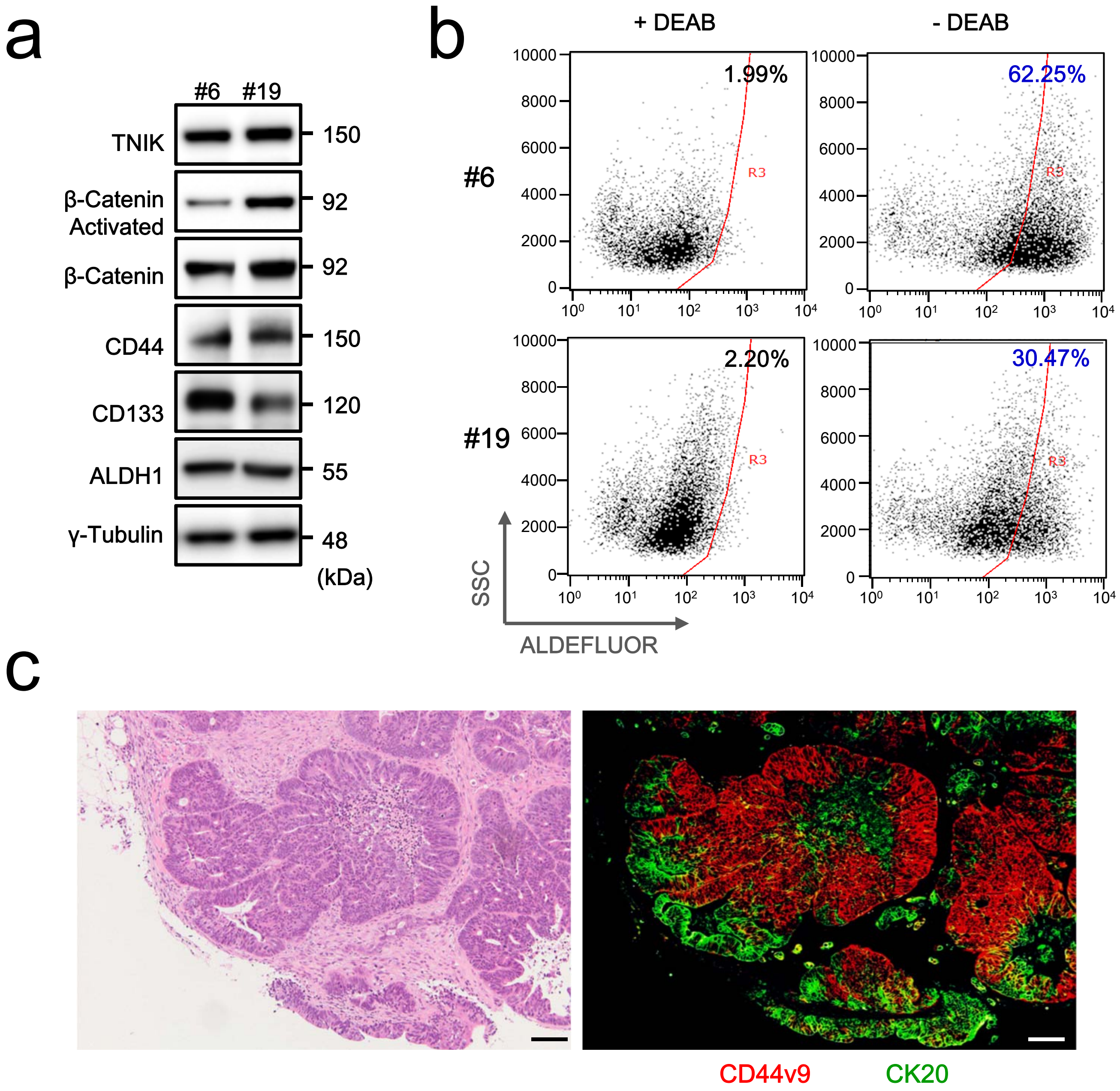

Supplementary Figure 9: Characterization of Patient-derived Spheroids.
(**a**) Immunoblot analysis of activated β-catenin, β-catenin, CSC marker (CD44, CD133, ALDH1), and γ-tubulin (loading control) expression in patient-derived spheroids #6 and #19.
(**b**) Flow cytometry-based assay showing the ALDH activity of #6 and #19 spheroids in the presence (+) and absence (-) of an ALDH inhibitor, DEAB. The number in each panel reflects the percentage of cells with ALDH activity.
(**c**) Representative histology and the CD44v9 (red) and CK20 (green) expression of a tumor established by transplanting patient-derived spheroids #6 into an immunodeficient mouse. Bars, 100 μm.

a

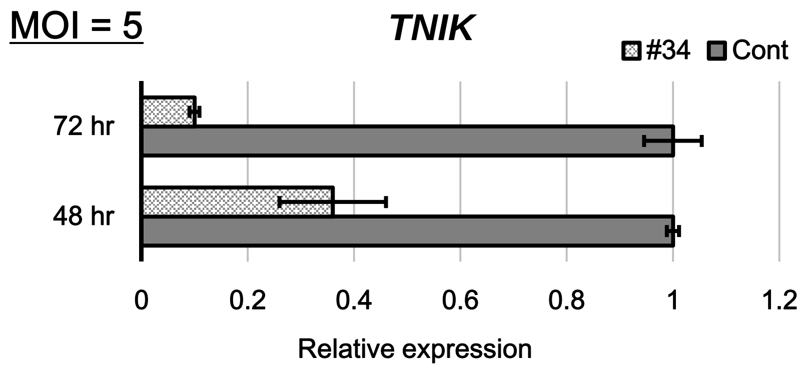

b

48 hr

		Sphere-containing wells/total wells				Sphere-initiating cell frequency		Fold reduction	Probability
		Cells per well							
		1000	100	10	1	Estimate (95% confidence interval)			
MOI = 5	Cont.	96/96	96/96	67/96	8/96	1/8.7	(1/11-1/6.9)		
	#34	96/96	96/96	50/96	3/96	1/14.46	(1/18.7-1/11.2)	1.7	***

*** *P* < 0.001

72 hr

		Sphere-containing wells/total wells				Sphere-initiating cell frequency		Fold reduction	Probability
		Cells per well							
		1000	100	10	1	Estimate (95% confidence interval)			
MOI = 5	Cont.	96/96	96/96	63/96	6/96	1/9.9	(1/12.7-1/7.8)		
	#34	96/96	96/96	22/96	1/96	1/28.3	(1/35.9-1/22.3)	2.9	***

*** *P* < 0.001

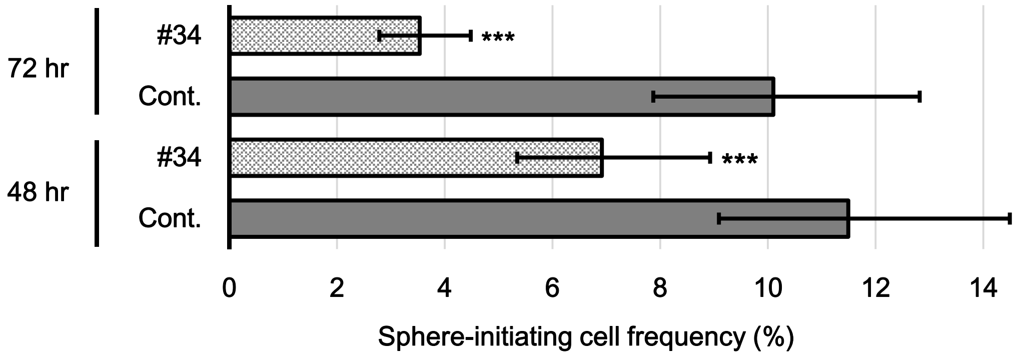

Supplementary Figure 10: Inhibition of Sphere Reformation by TNIK Knockdown.
(**a**) Patient-derived colorectal cancer spheroids #6 were infected with a lentiviral vector expressing shRNA for TNIK (#34) or the corresponding negative control vector (Cont). The expression level of *TNIK* was determined by real-time RT-PCR 48 or 72 hours later. Abbreviation: MOI, multiplicity of infection.
(**b**) Forty-eight or 72 hours after viral infection, the spheroids were gently dissociated into single cells, distributed into 96-well low-attachment cell culture clusters at a density of 1, 10, 100, or 1,000 cells per well, and cultured for 10 days. The frequency of sphere-reforming cells was estimated using ELDA software (***P < 0.001 relative to DMSO).

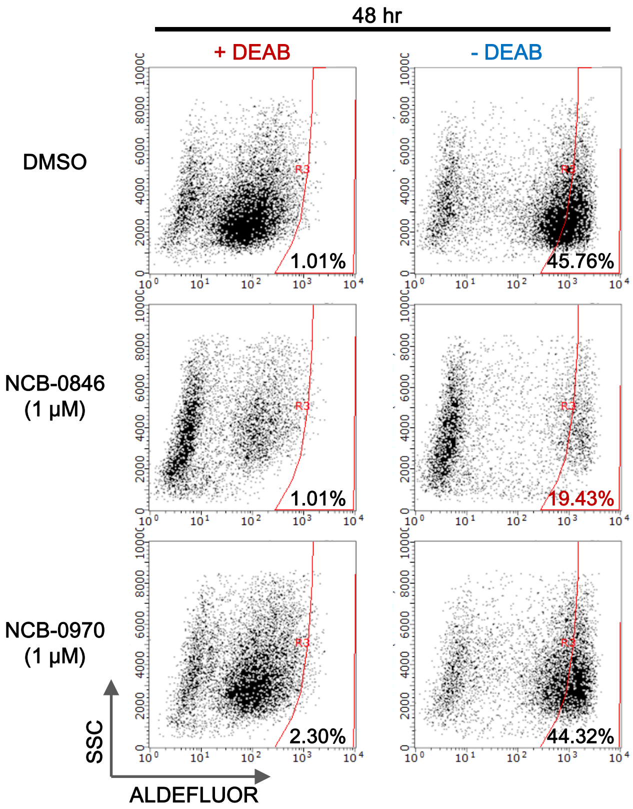

Supplementary Figure 11: Inhibition of Spheroid ALDH Activity.
Flow cytometry-based assay showing the percentage of cells with ALDH activity in spheroid #6 treated with DMSO (control), 1 μM NCB-0846, or 1 μM NCB-0970 for 48 hours.

3-day Treatment

		Sphere-containing wells/total wells				Sphere-initiating cell frequency		Fold reduction	Probability
		Cells per well							
		1000	100	10	1	Estimate (95% confidence interval)			
DMSO		96/96	96/96	84/96	12/96	1/5.25	(1/6.6-1/4.2)		
NCB-0846	0.1 μM	96/96	96/96	77/96	12/96	1/6.4	(1/8.0-1/5.1)	1.21	
	0.3 μM	96/96	96/96	72/96	3/96	1/8.3	(1/10.5-1/6.6)	1.57	***
	1.0 μM	96/96	94/96	49/96	2/96	1/18.5	(1/24-1/14.3)	3.49	***
NCB-0970	0.1 μM	96/96	96/96	83/96	8/96	1/5.7	(1/7.2-1/4.6)	1.08	
	0.3 μM	96/96	96/96	87/96	10/96	1/4.9	(1/6.2-1/3.9)	0.92	
	1.0 μM	96/96	96/96	50/96	1/96	1/15	(1/19.5-1/11.6)	2.83	***

*** $P < 0.001$

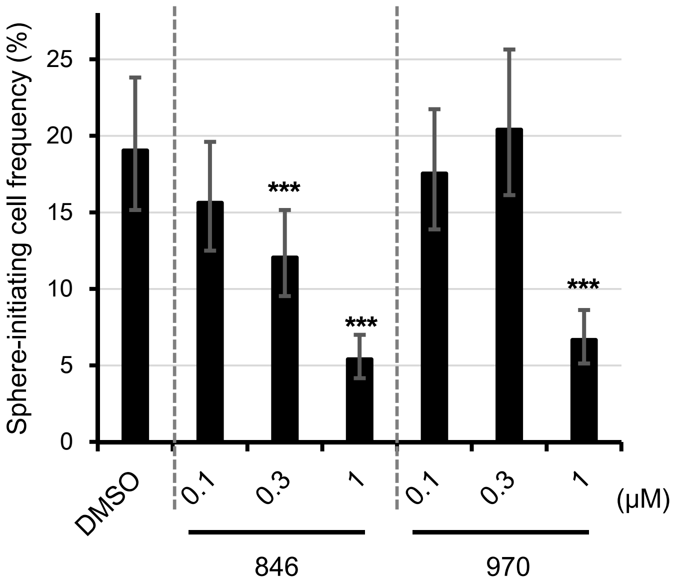

Supplementary Figure 12: Inhibition of Sphere Reformation by NCB-0846. Patient-derived colorectal cancer spheroids #6 were treated with DMSO or the indicated concentration of NCB-0846 or NCB-0970 for 72 hours. The spheroids were gently dissociated into single cells, distributed into 96-well low-attachment cell culture clusters at a density of 1, 10, 100, or 1,000 cells per well, and cultured for 10 days. The frequency of sphere-reforming cells was estimated using ELDA software (***$P < 0.001$ relative to DMSO).

COX021: PDX established from a 57-year old male patient

Histology: Poorly differentiated adenocarcinoma of the colon

a

Primary tumor | PDX

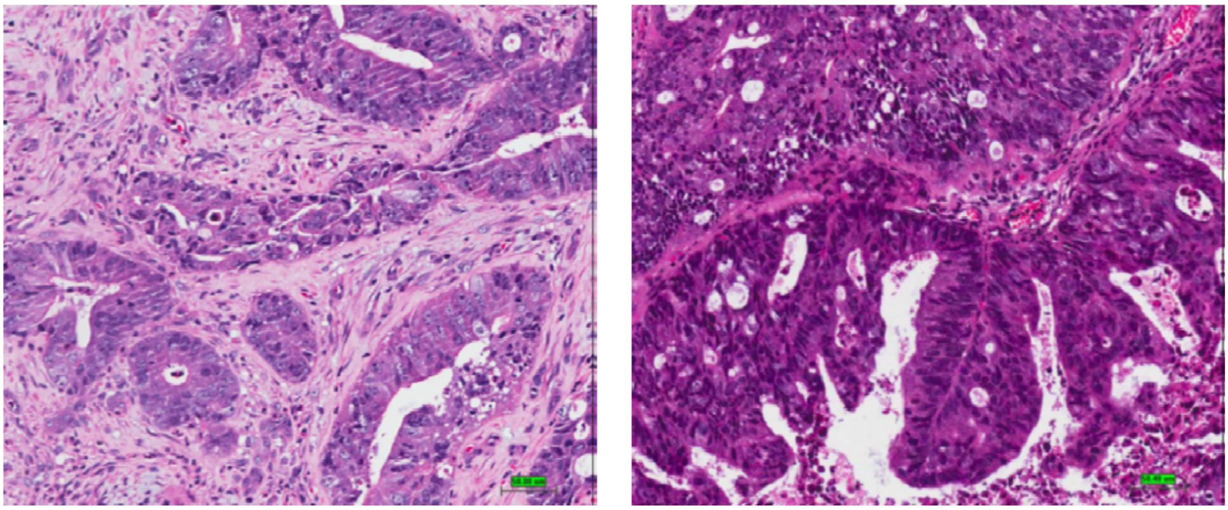

b

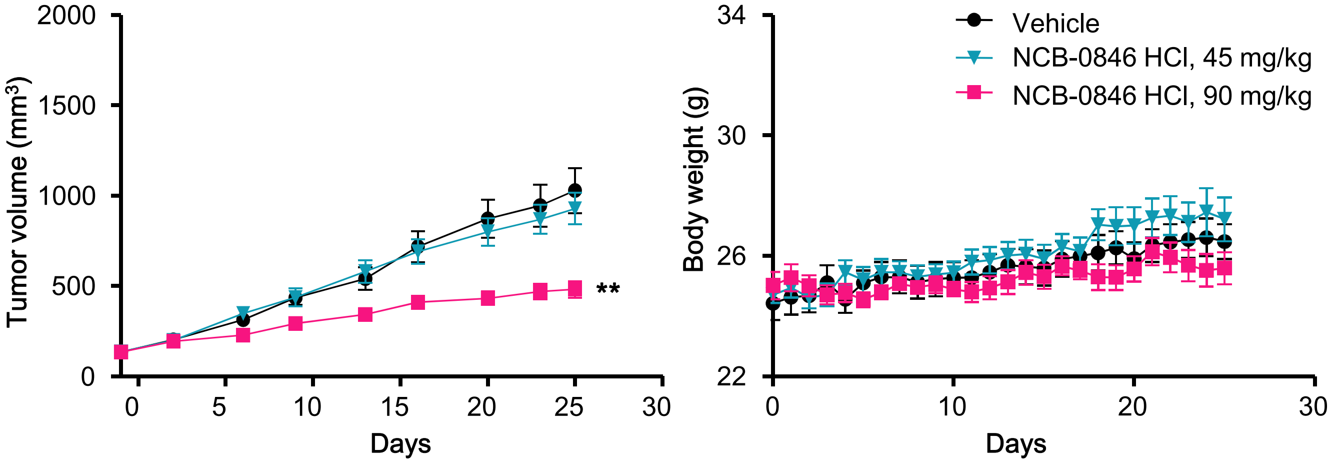

Supplementary Figure 13: Anti-tumor Activity of NCB-0846 against PDX (COX021).
(**a**) Histology of the primary tumor (left) and a xenograft (right) established from the same patient. Scale bars, 50 µm.
(**b**) PDXs were transplanted into the subcutaneous tissues of female 5-6-week-old Nu/Nu nude mice. When the volume of tumors reached ~200 mm^3, oral administration of 0 (vehicle alone, *n* = 10), 45 (*n* = 10) or 90 (*n* = 10) mg/kg NCB-0846 HCl (hydrochloride salt of NCB-0846) was initiated using a 5-day on, 2-day off BID schedule. $^{**}P < 0.01$.

COX026: PDX established from a 66-year old female patient

Histology: Poorly differentiated adenocarcinoma of the colon

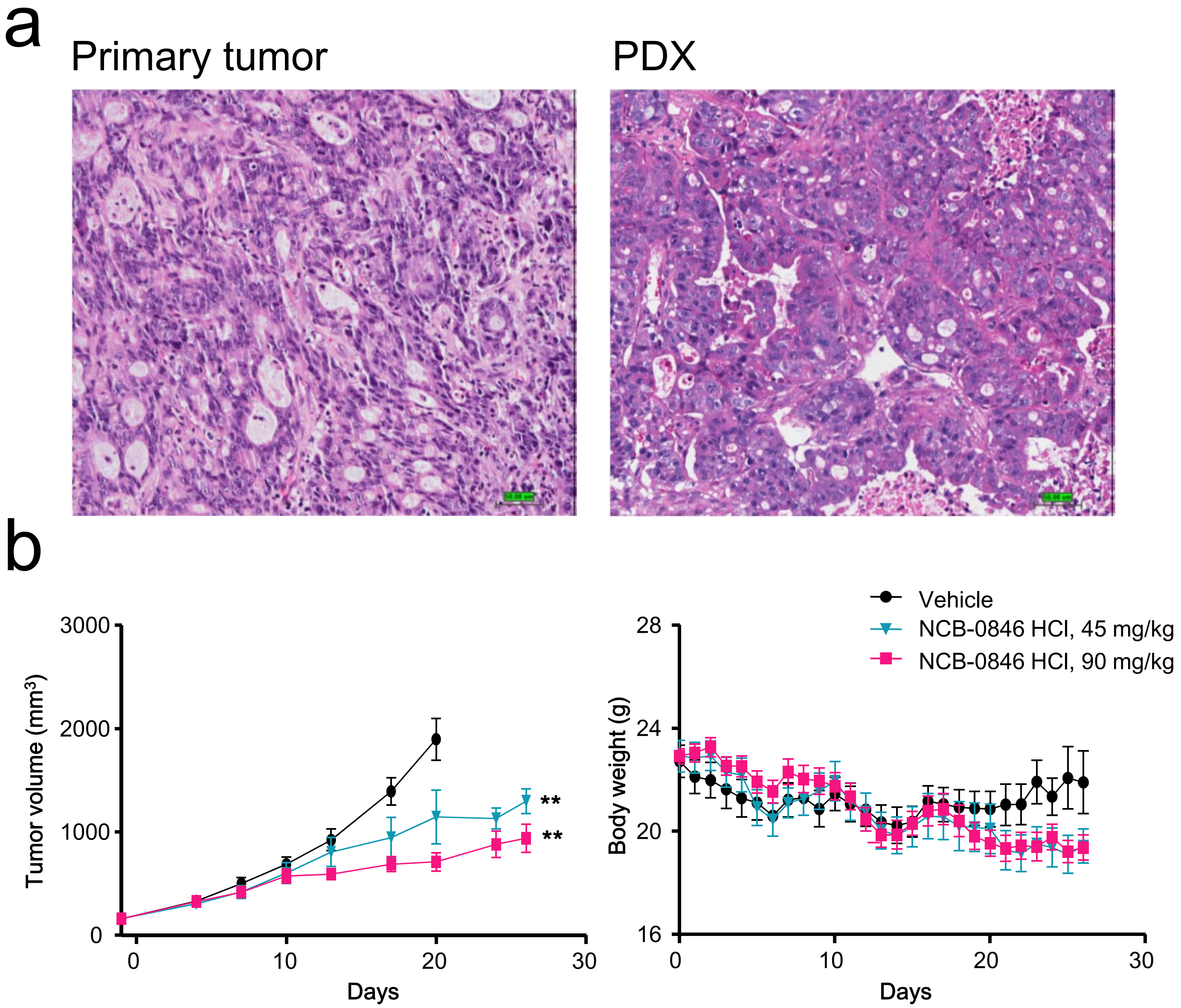

Supplementary Figure 14: Anti-tumor Activity of NCB-0846 against PDX (COX026).
(**a**) Histology of the primary tumor (left) and a xenograft (right) established from the same patient. Scale bars, 50 µm.
(**b**) PDXs were transplanted into the subcutaneous tissues of female 5-6-week-old Nu/Nu nude mice. When the volume of tumors reached ~200 mm^3, oral administration of 0 (vehicle alone, n = 10), 45 (n = 10) or 90 (n = 10) of NCB-0846 HCl was initiated using a 5-day on, 2-day off BID schedule. Some mice in the vehicle group (blue line) were euthanatized on day 20, as their tumors exceeded the predetermined ethical criteria. $^{**}P <$ 0.01.

a

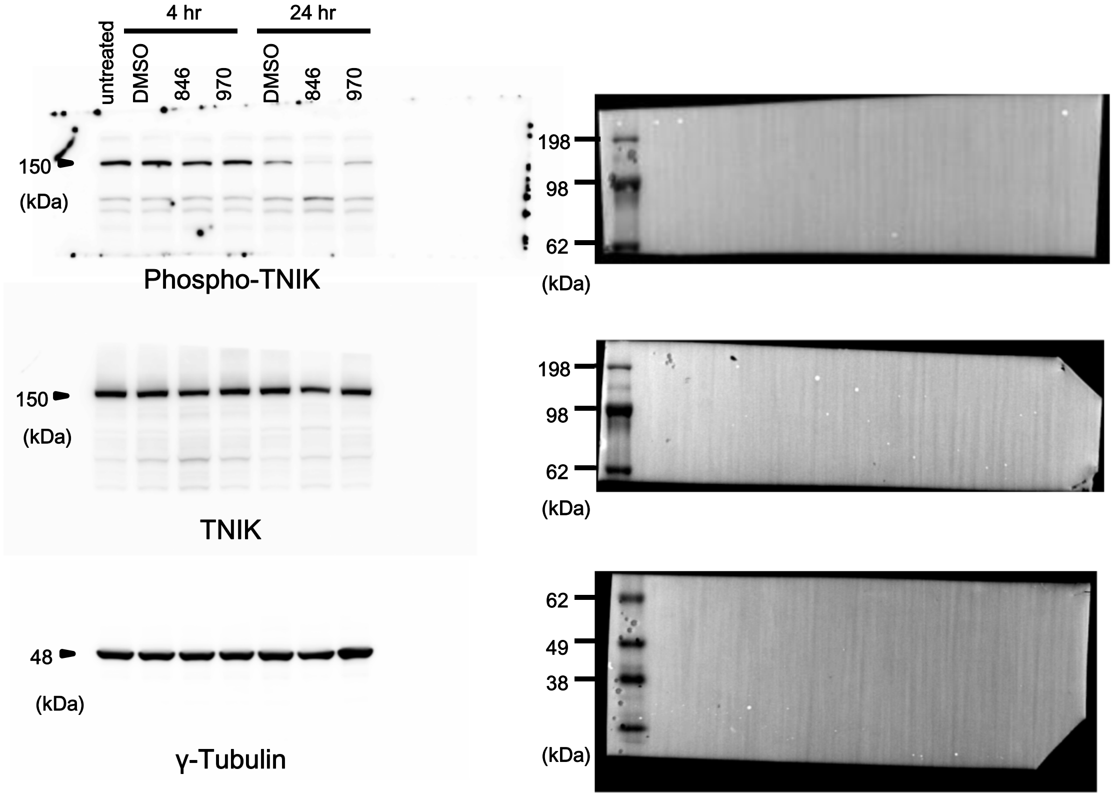

b

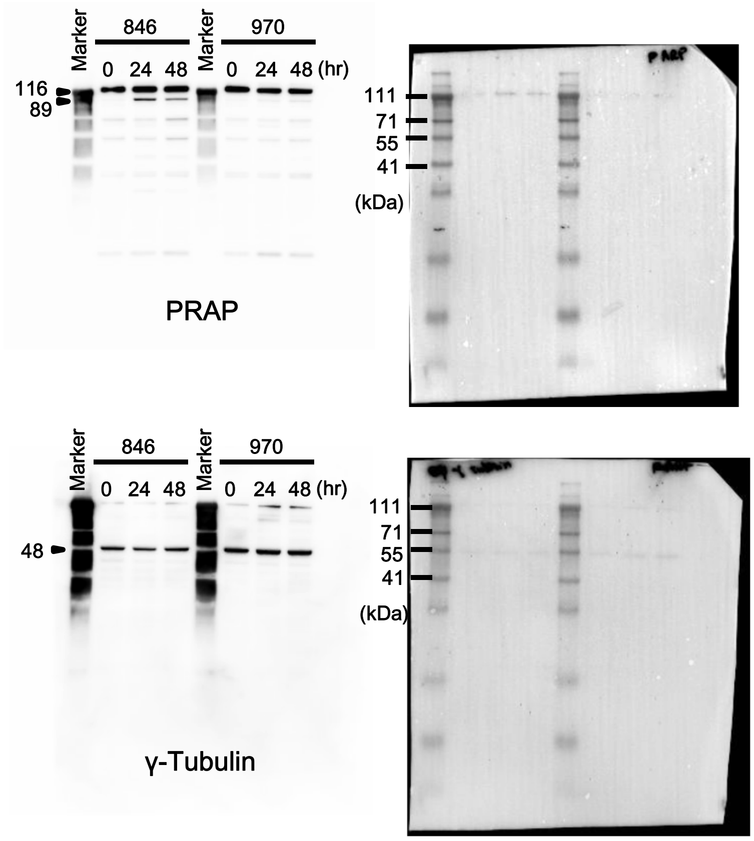

Supplementary Figure 15: Uncropped versions of the blots in Figures 2e (a) and 3f (b).

Supplementary Table 1. **Selectivity Profiles of NCB-0846 and NCB-0970.**

Kinases	Abbreviations	NCB-0846	NCB-0970
c-Abl oncogene 1	ABL	16.9	3.9
c-Src tyrosine kinase	CSK	-1.0	-4.0
Epidermal growth factor receptor	EGFR	4.8	-16.8
EPH receptor A2	EPHA2	13.6	8.7
EPH receptor A4	EPHA4	5.5	2.3
Fibroblast growth factor receptor 1	FGFR1	32.5	14.4
Fms-related tyrosine kinase 3	FLT3	104.1	101.7
Insulin-like growth factor 1 receptor	IGF1R	10.7	1.2
IL2-inducible T-cell kinase	ITK	10.0	9.0
Janus kinase 3	JAK3	96.3	85.4
Kinase insert domain receptor	KDR	71.1	43.7
Lymphocyte-specific protein tyrosine kinase	LCK	23.6	6.8
Hepatocyte growth factor receptor	MET	42.1	16.1
Platelet-derived growth factor receptor α	PDGFRα	89.0	45.6
Proline-rich tyrosine kinase 2	PYK2	73.0	23.6
v-Src sarcoma viral oncogene homolog	SRC	19.0	14.4
Spleen tyrosine kinase	SYK	7.6	14.2
TEK tyrosine kinase	TIE2	4.9	-2.8
Tropomyosin receptor kinase A	TRKA	89.1	81.6
Tyro3 receptor tyrosine kinase	TYRO3	48.9	23.9
v-Akt murine thymoma viral oncogene homolog 1	AKT1	-3.4	-4.4
AMP-activated protein kinase	AMPKα1/β1/γ1	31.5	9.3
Aurora kinase A	AurA	41.4	36.5
Calcium/calmodulin-dependent protein kinase IV	CaMK4	-1.5	-4.1
Cyclin-dependent kinase 2/cyclin A2	CDK2/CycA2	101.1	93.0
Checkpoint kinase 1	CHK1	10.1	9.0
Casein kinase 1ε	CK1ε	-2.8	-1.6
Death-associated protein kinase 1	DAPK1	3.7	4.7
Dual-specificity tyrosine-phosphorylation regulated kinase 1B	DYRK1B	8.9	-2.4
Mitogen-activated protein kinase 1	Erk2	0.7	-2.1
Glycogen synthase kinase 3β	GSK3β	27.7	13.5
Hematopoietic progenitor kinase	HGK	80.5	21.8
I-kappa-B kinase-β	IKKβ	6.8	7.9
Interleukin 1 receptor-associated kinase 4	IRAK4	14.7	6.3
c-Jun kinase 2	JNK2	0.3	-3.0
Mitogen-activated protein kinase-activated protein kinase 2; mapkapk2	MAPKAPK2	-5.8	-9.3
Macrophage stimulating 1	MST1	57.8	14.3

Never in mitosis gene A-related kinase 2	NEK2	-3.5	-4.5
Mitogen-activated protein kinase 14	p38α	-3.5	-2.8
Ribosomal protein S6 kinase	p70S6K	11.0	11.4
P21 protein (Cdc42/Rac)-activated kinase 2	PAK2	-7.8	-17.4
PDZ binding kinase	PBK	-3.9	-5.8
Pyruvate dehydrogenase kinase, isozyme 1	PDK1	2.4	-0.8
Pim-1 oncogene	PIM1	-3.1	-5.0
cAMP-dependent protein kinase, catalytic subunit α	PKACα	0.9	-4.4
Protein kinase C-α	PKCα	-0.9	1.4
Polycystic kidney disease 2	PKD2	18.8	1.0
Rho-associated, coiled-coil containing protein kinase 1	ROCK1	20.1	9.7
Serum/glucocorticoid regulated kinase	SGK	14.7	6.6
Testis-specific serine kinase 1	TSSK1	5.8	1.1

Percentage inhibition was determined at an inhibitor concentration of 0.1 μM with ATP at the Km concentration.

Supplementary Table 2. Reduction in the Frequency of Sphere-forming Cells by NCB-0846.

HCT116

3-day Treatment

		Sphere-containing wells/total wells			Sphere-initiating cell frequency			
		Cells per well						
		100	10	1	Estimate (95% confidence interval)		Fold reduction	Probability
DMSO		96/96	96/96	62/96	1/1.6	(1/1.8 - 1/1.4)		
NCB-0846	0.1μM	96/96	96/96	60/96	1/1.6	(1/1.9 - 1/1.4)	1.0	
	0.3μM	96/96	96/96	47/96	1/2.0	(1/2.5 - 1/1.7)	1.3	*
	1.0μM	95/96	21/96	3/96	1/30.1	(1/37.9 - 1/23.9)	18.8	***
NCB-0970	0.1μM	96/96	96/96	62/96	1/1.6	(1/1.8 - 1/1.4)	1.0	
	0.3μM	96/96	96/96	65/96	1/1.5	(1/1.7 - 1/1.3)	0.9	
	1.0μM	96/96	96/96	60/96	1/1.6	(1/1.9 - 1/1.4)	1	

* $P < 0.05$, *** $P < 0.001$

4-day Treatment

		Sphere-containing wells/total wells			Sphere-initiating cell frequency			
		Cells per well						
		100	10	1	Estimate (95% confidence interval)		Fold reduction	Probability
DMSO		96/96	96/96	60/96	1/1.6	(1/1.9 - 1/1.4)		
NCB-0846	0.1μM	96/96	96/96	69/96	1/1.4	(1/1.6 - 1/1.2)	0.9	
	0.3μM	96/96	96/96	41/96	1/2.3	(1/2.8 - 1/1.9)	1.4	***
	1.0μM	93/96	19/96	0/96	1/35.9	(1/44.9 - 1/28.7)	22.4	***
NCB-0970	0.1μM	96/96	96/96	59/96	1/1.6	(1/1.9 - 1/1.4)	1.0	
	0.3μM	96/96	96/96	60/96	1/1.6	(1/1.9 - 1/1.4)	1.0	
	1.0μM	96/96	96/96	56/96	1/1.7	(1/2.0 - 1/1.5)	1.1	

*** $P < 0.001$

DLD-1

3-day Treatment

		Sphere-containing wells/total wells			Sphere-initiating cell frequency			
		Cells per well						
		100	10	1	Estimate (95% confidence interval)		Fold reduction	Probability
DMSO		96/96	96/96	50/96	1/1.9	(1/2.3- 1/1.6)		
NCB-0846	0.1 μM	96/96	95/96	55/96	1/1.9	(1/2.3 - 1/1.6)	1.0	
	0.3 μM	96/96	86/96	23/96	1/4.7	(1/5.8 - 1/3.9)	2.5	***
	1.0 μM	77/96	25/96	3/96	1/53.1	(1/66.1 - 1/42.7)	27.9	***
NCB-0970	0.1 μM	96/96	96/96	40/96	1/2.3	(1/2.9 - 1/1.9)	1.2	
	0.3 μM	96/96	95/96	51/96	1/2.1	(1/2.5 - 1/1.7)	1.1	
	1.0 μM	95/96	75/96	16/96	1/8.1	(1/10.1 - 1/6.6)	4.2	***

*** $P < 0.001$

4-day Treatment

		Sphere-containing wells/total wells			Sphere-initiating cell frequency			
		Cells per well						
		100	10	1	Estimate (95% confidence interval)		Fold reduction	Probability
DMSO		96/96	96/96	46/96	1/2.1	(1/2.5 - 1/1.7)		
NCB-0846	0.1 μM	96/96	92/96	39/96	1/3.0	(1/3.8 - 1/2.5)	1.5	
	0.3 μM	95/96	90/96	46/96	1/4.2	(1/5.1 - 1/3.5)	2	***
	1.0 μM	96/96	34/96	7/96	1/20.2	(1/26.0 - 1/15.7)	9.8	***
NCB-0970	0.1 μM	96/96	95/96	55/96	1/1.9	(1/2.3 - 1/1.6)	0.9	
	0.3 μM	96/96	94/96	65/96	1/1.8	(1/2.1 - 1/1.5)	0.9	
	1.0 μM	96/96	95/96	45/96	1/2.3	(1/2.8 - 1/1.9)	1.1	

*** $P < 0.001$

Supplementary Table 3. Real-time PCR Primer and Probe Sets Used in This Study.

Figure		Gene symbol	Species	Resource	Catalog #
Figure 1	b	*Tnik*	mouse	Applied Biosystems	Mm01286435_m1
		Actb1	mouse	Applied Biosystems	4352933
	d	*Tnik*	mouse	Applied Biosystems	Mm01286435_m1
		Axin2	mouse	Applied Biosystems	Mm00443610_m1
		Myc	mouse	Applied Biosystems	Mm00487804_m1
		Cd44	mouse	Applied Biosystems	Mm01277163_m1
		Actb1	mouse	Applied Biosystems	4352933
Figure 2	h	*TNIK*	human	Applied Biosystems	Hs00323234_m1
		AXIN2	human	Applied Biosystems	Hs00610344_m1
		CCND1	human	Applied Biosystems	Hs00277039_m1
		MYC	human	Applied Biosystems	Hs00153408_m1
		ACTB	human	Applied Biosystems	Hs99999903_m1
Figure 3	d	*AXIN2*	human	Applied Biosystems	Hs00610344_m1
		MYC	human	Applied Biosystems	Hs00153408_m1
		CCND1	human	Applied Biosystems	Hs00277039_m1
		ACTB	human	Applied Biosystems	Hs99999903_m1
Supplementary Figure S2	b	*Mink1*	mouse	Applied Biosystems	Mm00489533_m1
		Nrk	mouse	Applied Biosystems	Mm00479081_m1
		Map4k4	mouse	Applied Biosystems	Mm00500812_m1
		Actb1	mouse	Applied Biosystems	4352933
Supplementary Figure S10	a	*TNIK*	human	Applied Biosystems	Hs00323234_m1
		ACTB	human	Applied Biosystems	Hs99999903_m1

Supplementary Table 4. Antibodies Used in This Study.

Figure		Application	Antigen	Antibody	Conjugated with	Clone	Resource	Catalog #	Dilution(1:)
Figure 2	d	IB	pSerine	Rabbit polyclonal			Thermo Fisher Scientific	61-8100	200
		IB	TCF4	Rabbit monoclonal		C48H11	CST	2569	1000
	e	IB	pTNIK Ser764	Rabbit monoclonal		#35	in-house		200
		IB	TNIK	Mouse monoclonal		53	BD	612250	2000
		IB	γ-Tubulin	Mouse monoclonal		GTU-88	SIGMA	T6557	1000
	i	IB	TNIK	Mouse monoclonal		53	BD	612250	2000
		IB	AXIN2	Rabbit monoclonal		76G6	CST	2151	1000
		IB	cMYC	Rabbit monoclonal		D84C12	CST	5605	1000
		IB	LRP6	Rabbit monoclonal		C5C7	CST	2560	1000
		IB	LRP5	Rabbit monoclonal		D80F2	CST	5731	1000
		IB	β-Actin	Mouse monoclonal		AC-15	Abcam	ab6276	10000
Figure 3	f	IB	PARP	Rabbit polyclonal			CST	9542	1000
		IB	γ-Tubulin	Mouse monoclonal		GTU-88	SIGMA	T6557	1000
Figure 5	a	IB	CD44	Mouse monoclonal		156-3C11	Thermo Fisher Scientific	MS668P0	500
		IB	CD133/1	Mouse monoclonal		W6B3C1	Miltenyi Biotec	130-092-395	100
		IB	ALDH1A1	Rabbit monoclonal		D4R9V	CST	12035	1000
		IB	TNIK	Mouse monoclonal		53	BD	612250	2000
		IB	γ-Tubulin	Mouse monoclonal		GTU-88	SIGMA	T6557	1000
		IB	Slug	Rabbit monoclonal		C19G7	CST	9585	1000
		IB	Snail	Rabbit monoclonal		C15D3	CST	3879	1000
		IB	Twist	Mouse monoclonal		2C1a	Abcam	ab50887	50
		IB	Smad2	Rabbit monoclonal		D43B4	CST	5339	1000
		IB	Vimentin	Rabbit monoclonal		D21H3	CST	5741	1000
		IB	E-cadherin	Mouse monoclonal		34	BD	610404	250
		IB	Oct-4A	Rabbit monoclonal		C30A	CST	2840	1000
		IB	Nanog	Rabbit monoclonal		D73G4	CST	4903	2000
		IB	Sox2	Rabbit monoclonal		D6D9	CST	3579	1000
Figure 6	e	FC	CD133	Mouse IgG1	APC	AC133	Miltenyi Biotec	130-090-826	§
		FC	NA	Isotype-matched mouse IgG1	APC	IS5-21F5	Miltenyi Biotec	130-092-214	§
		FC	CD44	Mouse IgG2b,κ	PE	G44-26 (C26)	BD Pharmingen	555479	§
		FC	NA	Isotype-matched mouse IgG2b,κ	PE	27-35	BD Pharmingen	555743	§
		FC	CD24	Mouse IgG2a,κ	Alexa 647	ML5	BioLegend	311110	§
		FC	NA	Isotype-matched mouse IgG2a,κ	Alexa 647	MOPC-173	BioLegend	400234	§
		FC	CD166	Mouse IgG1	PE	3A6	Beckman Coulter	A22361	§
		FC	NA	Isotype-matched mouse IgG1	PE	679.1Mc7	Beckman Coulter	A07796	§
		FC	CD29	Mouse IgG1,κ	Alexa 647	TS2/16	BioLegend	303018	§
		FC	NA	Isotype-matched mouse IgG1,κ	Alexa 647	MOPC-21	BioLegend	400130	§
		FC	EpCAM	Mouse IgG2b,κ	PE	9C4	BioLegend	324206	§
		FC	NA	Isotype-matched mouse IgG2b,κ	PE	MOPC-21	BioLegend	400314	§
	g	IHC	Ki67	Mouse monocolnal		MM1	Leica biosystems	NCL-L-Ki67-MM1	100
		IHC	CD44	Mouse monoclonal		156-3C11	Thermo Fisher Scientific	MS668P0	300
Supplementary Figure S7		FC	CD133	Mouse IgG1	APC	AC133	Miltenyi Biotec	130-090-826	§
		FC	NA	Isotype-matched mouse IgG1	APC	IS5-21F5	Miltenyi Biotec	130-092-214	§
		FC	CD44	Mouse IgG2b,κ	PE	G44-26 (C26)	BD Pharmingen	555479	§
		FC	NA	Isotype-matched mouse IgG2b,κ	PE	27-35	BD Pharmingen	555743	§
		FC	CD24	Mouse IgG2a,κ	Alexa 647	ML5	BioLegend	311110	§
		FC	NA	Isotype-matched mouse IgG2a,κ	Alexa 647	MOPC-173	BioLegend	400234	§
		FC	CD166	Mouse IgG1	PE	3A6	Beckman Coulter	A22361	§
		FC	NA	Isotype-matched mouse IgG1	PE	679.1Mc7	Beckman Coulter	A07796	§
		FC	CD29	Mouse IgG1,κ	Alexa 647	TS2/16	BioLegend	303018	§
		FC	NA	Isotype-matched mouse IgG1,κ	Alexa 647	MOPC-21	BioLegend	400130	§
		FC	EpCAM	Mouse IgG2b,κ	PE	9C4	BioLegend	324206	§
		FC	NA	Isotype-matched mouseIgG2b,κ	PE	MOPC-21	BioLegend	400314	§
Supplementary Figure S9	a	IB	TNIK	Mouse monoclonal		53	BD	612250	2000
		IB	Active β-catenin	Mouse monoclonal		8E7	Merck Millipore	05-665	500
		IB	β-Catenin	Mouse monoclonal		14	BD	610153	500
		IB	CD44	Mouse monoclonal		156-3C11	Thermo Fisher Scientific	MS668P0	500
		IB	CD133/1	Mouse monoclonal		W6B3C1	Miltenyi Biotec	130-092-395	100
		IB	ALDH1A1	Rabbit monoclonal		D4R9V	CST	12035	1000
		IB	γ-Tubulin	Mouse monoclonal		GTU-88	SIGMA	T6557	1000
	c	IHC	CK20	Mouse monoclonal		Ks 20.8	Dako	M7019	250
		IHC	CD44 v9	Rat monocolonal		RV3	Cosmo Bio	LKG-M003	500
		IHC	Mouse IgG (H+L)	Goat polyclonal	Alexa 488		Thermo Fisher Scientific	A-11029	500
		IHC	Rat IgG (H+L)	Goat polyclonal	Alexa 568		Thermo Fisher Scientific	A-11077	500

§Concentrations recommended by manufacturers.

Abbreviation: IB, immunoblotting; FC, flow cytometry; IHC, immunohistochemistry; NA, not applicable.

Chemical synthesis

All commercial chemicals and solvents were reagent grade and used without further purification unless otherwise noted. Progress of the reactions was usually monitored by thin layer chromatography (TLC) using Merck silica gel 60 F254 plates. Column chromatography was performed using a RediSep® Normal-phase Silica Flash Column. 1H or 13C NMR spectra were recorded at 400 MHz (100 MHz for 13C) using a Bruker Avance III. Chemical shifts are reported in ppm with the internal TMS signal at 0.0 ppm as a standard. The data are reported as (s = singlet, d = doublet, t = triplet, q = quartet, m = multiplet, br = broad single, coupling constant(s) in Hz, integration). Analytical LCMS was performed on a Shimazu LCMS-2010A (Imtakt Cadenza CD-C18 column; 10 mM formic acid in water / 10 mM formic acid in acetonitrile gradient; UV detection at 215 and 254 nm) and mass spectra (MS) were recorded using electrospray ionization (ESI). High-resolution mass spectra (HRMS) were acquired on a Thermo Scientific Q Exactive hybrid quadrupole-Orbitrap mass spectrometer.

Synthesis of 2-(3H-benzoimidazol-5-ylamino)-quinazolin-8-ol (1)

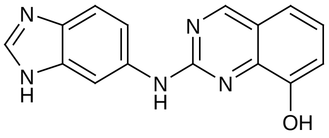

(**1**)

A mixture of 5-aminobenzimidazole (0.7 g, 5.26 mmol) and 2-chloro-quinazolin-8-ol (0.97 g, 5.26 mmol) in 2-propanol (15 mL) was refluxed for 16 hours. The reaction mixture was cooled to room temperature, and the resulting precipitates were collected by filtration. The solids were washed with 2-propanol to give 2-(3H-benzoimidazol-5-ylamino)-quinazolin-8-ol (1.07 g) as a brown solid.

Yield: 73%. 1H NMR (400 MHz, DMSO-d6) δ (ppm): 10.31 (s, 1H), 9.6 - 9.75 (br, 1H), 9.50 (s, 1H), 9.31 (s, 1H), 8.98 (d, J = 1.1 Hz, 1H), 7.96 (dd, J = 9.0, 1.7 Hz, 1H), 7.76 (d, J = 7.9 Hz, 1H), 7.42 (dd, J = 7.5, 1.1 Hz, 1H), 7.2 - 7.35 (m, 2H); LCMS (m/z) 278.2 $[M+H]^+$.

Synthesis of N-(1H-benzo[d]imidazol-6-yl)-8-[cis-4-(tert-butyldimethylsilyloxy)cyclohexyloxy]quinazolin-2-amine (2)

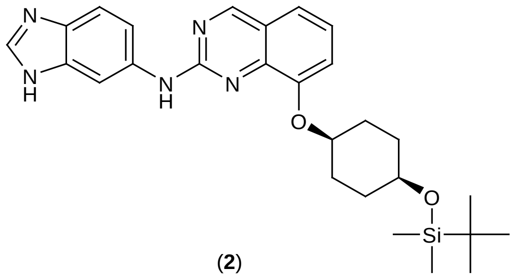

(**2**)

To a suspension of 2-(3H-benzoimidazol-5-ylamino)-quinazolin-8-ol (1.5 g, 5.41 mmol) and trans-4-(tert-butyldimethylsilyloxy)cyclohexyl methanesulfonate (2.5 g, 8.12 mmol) in DMSO was added cesium carbonate (5.45 g, 16.78 mmol), and the mixture was stirred at 130 °C for 1 h. The reaction mixture was diluted with ethyl acetate (50 mL) and THF (50 mL), and then diluted with ice-water (25 mL). The organic layer was separated and dried over sodium sulfate, and then concentrated under vacuum. The residue was purified by flash chromatography on silica (hexane/ethyl acetate, linear gradient) to obtain N-(1H-benzo[d]imidazol-6-yl)-8-[cis-4-(tert-butyldimethylsilyloxy)cyclohexyloxy]quinazolin-2-amine (1.16 g) as an oil.
Yield: 44%. 1H NMR (400 MHz, DMSO-d6) δ (ppm): 11.99 - 12.32 (m, 1H), 9.72 - 9.90 (m, 1H), 9.16 - 9.31 (m, 1H), 8.28 - 8.37 (m, 1H), 7.99 - 8.18 (m, 2H), 7.38 - 7.63 (m, 2H), 7.30 - 7.38 (m, 1H), 7.16 - 7.30 (m, 1H), 4.76 (br, 1H), 3.84 (br, 1H), 1.92 - 2.07 (m, 2H), 1.67 - 1.91 (m, 4H), 1.56 - 1.67 (m, 2H), 0.87 (s, 9H), 0.06 (s, 6H); LCMS (m/z) 490.2 $[M+H]^+$.

Synthesis of cis-4-[2-(1H-benzo[d]imidazol-6-ylamino)quinazolin-8-yloxy]cyclohexanol (NCB-0846)

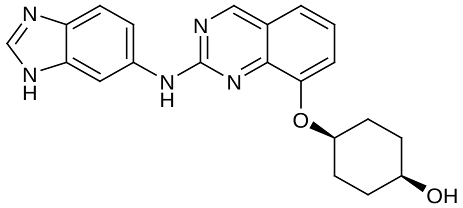

To a solution of N-(1H-benzo[d]imidazol-6-yl)-8-[cis-4-(tert-butyldimethylsilyloxy)cyclohexyloxy]quinazolin-2-amine (4.0 g, 8.18 mmol) in dioxane (20 mL) was added 4N HCl/dioxane (20 mL) at 0 °C, and the mixture was stirred at room temperature for 1 hour. The solvent was evaporated, and then saturated sodium bicarbonate solution was added to the residual oil. The resulting solids were collected

by filtration, and washed with ethyl acetate to give cis-4-[2-(1H-benzo[d]imidazol-6-ylamino)quinazolin-8-yloxy]cyclohexanol (2.0 g) as an off-white solid.
Yield: 65%, Purity: >99% by HPLC (UV at 215 and 254 nm). 1H NMR (400 MHz, DMSO-d6) δ (ppm): 12.29 - 12.54 (m, 1H), 9.73 - 10.0 (m, 1H), 9.15 - 9.3 (m, 1H), 8.65 - 9.11 (m, 1H), 7.98 - 8.27 (m, 1H), 7.52 - 7.69 (m, 2H), 7.4 - 7.52 (m, 1H), 7.36 (dd, J = 7.9, 1.2 Hz, 1H), 7.26 (t, J = 7.9 Hz, 1H), 5.51 - 5.9 (m, 1H), 4.88 (s, 1H), 3.45 - 3.85 (m, 1H), 1.82 - 2.13 (m, 4H), 1.49 - 1.85 (m, 4H); 13C NMR (100MHz, DMSO-d6) δ: 162.16, 156.61, 150.95, 143.95, 141.81, 136.34, 123.59, 121.68, 119.54, 116.32, 71.02, 68.56, 30.68, 28.07; HRMS (ESI): (m/z) calcd for $C_{21}H_{20}O_2N_5$ [M-H]- 374.1612, found 374.1608.

Synthesis of trans-4-[2-(1H-benzo[d]imidazol-6-ylamino)quinazolin-8-yloxy]cyclohexanol (NCB-0970)

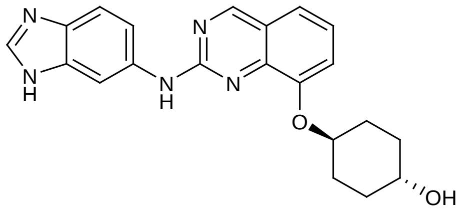

Trans-4-[2-(1H-benzo[d]imidazol-6-ylamino)quinazolin-8-yloxy]cyclohexanol (10.8 mg) was synthesized and purified using a procedure analogous to that for NCB-0846 from 2-(3H-benzoimidazol-5-ylamino)-quinazolin-8-ol and cis-4-(tert-butyldimethylsilyloxy)cyclohexyl methanesulfonate.
Yield: 5.6%, Purity: >99% by HPLC (UV at 215 and 254 nm). 1H NMR (400 MHz, DMSO-d6) δ (ppm): 12.10 - 12.37 (m, 1H), 9.71 - 10.00 (m, 1H), 9.11 - 9.34 (m, 1H), 8.27 - 8.72 (m, 1H), 8.03 - 8.20 (m, 1H), 7.69 - 7.91 (m, 1H), 7.29 - 7.60 (m, 3H), 7.13 - 7.31 (m, 1H), 4.56 - 4.73 (m, 1H), 4.46 - 4.56 (m, 1H), 3.55 - 3.75 (m, 1H), 2.02 - 2.22 (m, 2H), 1.83 - 1.99 (m, 2H), 1.53 - 1.71 (m, 2H), 1.23 - 1.43 (m, 2H); 13C NMR (100MHz, DMSO-d6) δ: 162.33, 156.76, 151.30, 144.49, 142.05, 136.17, 123.46, 121.79, 120.64, 119.33, 115.37,76.23, 67.61, 32.09, 28.78; HRMS (ESI): (m/z) calcd for $C_{21}H_{20}O_2N_5$ [M-H]- 374.1612, found 374.1608.
